# Zhen-Wu-Tang Induced Mitophagy to Protect Mitochondrial Function in Chronic Glomerulonephritis via PI3K/AKT/mTOR and AMPK Pathways

**DOI:** 10.3389/fphar.2021.777670

**Published:** 2021-12-21

**Authors:** Bihao Liu, Yiwen Cao, Dejuan Wang, Yuan Zhou, Peichun Zhang, Junbiao Wu, Junqi Chen, Jianguang Qiu, Jiuyao Zhou

**Affiliations:** ^1^ Department of Urology, The Sixth Affiliated Hospital of Sun Yat-Sen University, Guangzhou, China; ^2^ Guangdong Institute of Gastroenterology, The Sixth Affiliated Hospital of Sun Yat-Sen Univerisity, Guangzhou, China; ^3^ Department of Pharmacology, School of Pharmaceutical Sciences, Guangzhou University of Chinese Medicine, Guangzhou, China; ^4^ Department of Pharmacy, Zhongshan Jishuitan Orthp Aedic Hospital, Zhongshan, China; ^5^ The Second Affiliated Hospital, Guangzhou University of Chinese Medicine, Guangzhou, China

**Keywords:** chronic glomerulonephritis, Zhen-wu-tang, mitochondrial function, mitophagy, PI3K/Akt/mTOR pathway, AMPK pathway

## Abstract

Chronic glomerulonephritis (CGN) is one of the major causes of end-stage kidney disease. Zhen-wu-tang (ZWT), as a famous Chinese herbal prescription, is widely used in China for CGN therapy in clinic. However, the mechanism of ZWT in CGN has not been fully understood. The present study explored the therapeutic effect and the underlying mechanism of ZWT on mitochondrial function in cationic bovine serum albumin (C-BSA)-induced CGN model rats and tumor necrosis factor (TNF-α)-damaged mouse podocytes. The renal functions were measured by serum creatinine (Scr) and blood urea nitrogen (BUN). Renal pathological changes and ultrastructure of kidney tissues were evaluated by periodic acid-Schiff (PAS) staining and transmission electron microscopy. The levels of antioxidases, including mitochondrial catalase (CAT), superoxide dismutase 2 (SOD2), and peroxiredoxin 3 (PRDX3), in CGN rats were examined by real-time PCR. The mitochondrial functions of podocytes were measured by ATP concentration, mitochondrial membrane potential (MMP), and mitochondrial ROS (mtROS). For mitophagy level detection, the expressions of mitophagy-related proteins, including LC3, p62, heat shock protein 60 (HSP60), and translocase of outer mitochondrial membrane 20 (TOMM20), were measured by Western blot, as the colocation of LC3 and mitochondrial marker COX IV were evaluated by immunofluorescence. Our results manifested that ZWT ameliorated CGN model rats by a remarkable decrease in Scr and BUN, inhibition of mesangial matrix proliferation, protection against foot processes fusion, and basement membrane thickening. More importantly, ZWT protected against mitochondrial dysfunction by increasing the expressions of CAT, SOD2, and PRDX3 in CGN model rats, increased ATP content and MMP in podocytes, and decreased excessive mtROS. Furthermore, ZWT induced mitophagy in CGN through increasing the expression of LC3, and decreasing p62, HSP60, TOMM20, and ZWT also enhanced the colocation of LC3 to the mitochondria. We found that ZWT inhibited the PI3K/AKT/mTOR pathway, which could be disturbed by PI3K inhibitor LY294002 and agonist insulin-like growth factor 1. Moreover, ZWT reversed the inhibition of the AMPK pathway in CGN. Overall, ZWT ameliorated renal mitochondrial dysfunction probably by inducing mitophagy via the PI3K/AKT/mTOR and AMPK pathways.

## Introduction

In 2017, 1.2 million people died from chronic kidney disease (CKD), and the global all-age mortality rate of CKD increased by 41.5% within the last 28 years (Global, 2020). Chronic glomerulonephritis (CGN) leads to about 20% of CKD, with clinical presentations of proteinuria, hematuria, edema, and hypertension (Floege and Amann, 2016). As a progressive disorder, CGN is the most frequent cause of end-stage renal disease (ESRD) in some developing countries (Chadban and Atkins, 2005). Conservative management by inhibitors of the renin–angiotensin–aldosterone axis is the main approach for proteinuria reduction, but whether angiotensin-converting enzyme inhibitors or angiotensin receptor blockers should be used alone is controversial (Voskamp et al., 2017). In addition to supportive therapy, immunosuppressive agents are widely used in the management of CGN patients. However, many immunosuppressive agents have a narrow therapeutic window and need close monitoring to balance the risk and benefits (Jefferson, 2018). Emerging therapies undergoing clinical trials are testing with dual angiotensin receptor/endothelin receptor blockers, SGLT2 inhibition, and drugs targeting Nrf2 transcription factor (Ramos et al., 2020). To sum up, there is no effective treatment that can prevent CGN patients from developing ESRD (Zhang and Zuo, 2016). Therefore, it is of great importance to clarify the pathogenesis of CGN, which will help us improve the therapeutic effect of CGN and the prognosis of patients.

Zhen-wu-tang (ZWT), recorded in the *Treatise on Febrile Diseases*, is composed of five traditional Chinese medicines including *Aconitum carmichaelii* Debeaux, *Poria cocos* (Schw.) Wolf, *Atractylodes macrocephala* Koidz, *Paeonia lactiflora* Pall, and *Zingiber officinale* Roscoe (Li et al., 2020a). Notably, ZWT has been widely used in treating various kinds of CGN patients in China. Besides, our previous studies exhibited nephroprotective effects on cationic bovine serum albumin (C-BSA)-induced CGN model by inhibiting inflammation (Wu et al., 2016; Liu et al., 2019). However, the regulatory mechanisms of ZWT in CGN has not been fully explained.

Mitochondrial damage and dysfunction participate in the pathogenesis of kidney diseases, which not only damage the renal cells but also affect the infiltrating inflammatory cells in the kidneys (Galvan et al., 2017). Under the stimulation of reactive oxygen species (ROS), proteinuria, and other pathogenic factors, the mitochondria are prone to dysfunction, which is manifested as abnormal morphology and structure, decreased ATP synthesis, mitochondrial ROS (mtROS), mitochondrial DNA damage, and mitochondrial dynamic imbalance (Forbes and Thorburn, 2018). Mitophagy is a selective form of autophagy that can specifically remove damaged mitochondria. Recent studies have shown that mitophagy plays an important role in the occurrence and development of acute kidney injury (AKI) and CKD (Wang et al., 2020).

Recently, growing evidence has demonstrated that the phosphatidylinositol 3-kinase (PI3K)/AKT/mammalian target of rapamycin (mTOR) signaling pathway plays an indispensable role in renal diseases to regulate autophagy (Tu et al., 2019). PI3K is involved in the formation of eukaryotic cell membranes, regulating cell signal transduction, energy metabolism, and cell cycle and other intracellular processes. AKT is the downstream regulatory target of PI3K, which takes part in the regulation of cell cycle, autophagy, and apoptosis processes (Yudushkin, 2019). Mammalian target of rapamycin (mTOR), activated by AKT, is a key signal molecule that regulates autophagy (Gong et al., 2020). Rapamycin, inhibitor of mTOR, enhanced mitophagy and attenuated mitochondrial apoptosis after spinal ischemia–reperfusion injury (Li et al., 2018). However, the relationship between the PI3K/AKT/mTOR pathway and mitophagy in CGN is unclear. Recent studies found that the AMP-activated protein kinase (AMPK) agonist alleviated renal tubulointerstitial fibrosis via activating mitophagy in diabetic mice (Han et al., 2021). In this study, the potential mechanism of ZWT on mitochondrial functions related to mitophagy in CGN was investigated.

## Materials and Methods

### Herbal Material and Zhen-wu-tang Preparation

The herbal material of *Aconitum carmichaelii* Debeaux (Fuzi), *Poria cocos* (Schw.) Wolf (Fu ling), *Atractylodes macrocephala* Koidz (Bai zhu), *Paeonia lactiflora* Pall (Bai shao), and *Zingiber officinale* Roscoe (Sheng jiang) was purchased from Guangzhou Caizhilin Pharmaceutical Co., Ltd. (Guangzhou, China), which constitutes ZWT in the ratio of 3:3:3:2:3. According to clinical usage, the daily intragastric doses were 4.2 kg/kg, 8.4 g/kg, 16.8 g/kg for low, middle, and high doses of ZWT (ZWT-L, ZWT-M, ZWT-H), respectively. The water extract of ZWT was prepared as described previously (Liu et al., 2019). Briefly, the materials were soaked with 10 times distilled water for 30 min and then boiled for 2 h. Subsequently, the medicinal residue was boiled again eight times with distilled water for 1.5 h. Finally, the filtrates above were concentrated to 1.68 g/ml based on raw materials.

### UPLC-Q-TOF-MS Analysis of Zhen-wu-tang

The extract of ZWT was used for UPLC-Q-TOF-MS analysis according to our previous study (Liang et al., 2019). The chromatographic separation was achieved by Shimadzu UPLC-30AD (Shimadzu Corporation, Kyoto, Japan) equipped with a Phenomenex Genmini 3u-C18-110 column (150 × 2 mm, 3 μm). The mobile phases included 0.025% formic acid in water (A) and acetonitrile (B) at an ambient temperature of 35°C. The linear gradient elution is as follows: 0–10 min, 5% B; 10–12 min, 8% B; 12–15 min, 20% B; 15–30 min, 35% B; 30–35 min, 45% B; 35–45 min, 95% B. The flow rate was set at 0.3 ml/min. The sample injection volume was 5 μl. Mass spectrometry was measured by the ABsciex Triple TOF 5600 mass spectrometer (ABsciex, Framingham, MA, USA), and the data were analyzed using Peakview software (Version 2.0, ABsciex). The mass parameters were as follows: ion source in electrospray mode: negative; electrospray ionization: 55 psi; IonSpray Voltage Floating: 5,500 V; declustering potential: 100 V; collision energy: 45 eV.

### Animals

Male Sprague–Dawley rats weighting 180–220 g were provided and housed in the Experimental Animal Center of Guangzhou University of Traditional Chinese Medicine. During the experiment, all rats were maintained at 25 ± 2°C and 55–65% humidity in special pathogen-free level. The animals had free access to food and fresh water. All animal experiment procedures were conducted in accordance with the guidelines of the Animal Ethics Committee of Guangzhou University of Chinese Medicine. All efforts were made to minimize the suffering of the animals.

### Experimental Protocols

The CGN rat model was established with C-BSA as we described before (Lu et al., 2020). Briefly, rats were subcutaneously injected with 1-ml emulsion including 1 mg of C-BSA and 0.5 ml of Freund’s incomplete adjuvant (Thermo Fisher Scientific, Boston, MA, USA) on the first day. From the 7th day to the 28th day, the model rats were injected intravenously in tail vein with 2.5 mg of C-BSA every other day. Afterward, 40 CGN model rats were picked and randomly divided into five groups including the model group, ZWT-L group, ZWT-M group, ZWT-H group, and prednisone group, with eight rats in each group. As clinical medicine for CGN therapy, prednisone was carried as the positive control. Except for the rats in the control and model group, the rats were orally administrated with different doses of ZWT or 2 mg/kg of prednisone acetate (Guangdong Huanan Pharmaceutical, Dongguan, China), respectively, for 4 weeks. The rats in the control and model groups were given 10 ml/kg of saline at the same time.

### Biochemical Analysis of Blood

When the interventions ended, blood of all rats obtained from the abdominal aorta were centrifuged at 3,500 rpm at room temperature for 15 min. Nest, serum creatinine (Scr), and blood urea nitrogen (BUN) were measured according to the instruction of the manufacturer (Jiancheng Bioengineering Institute, Nanjing, China).

### Periodic Acid-Schiff Staining

Kidney tissues of each group were fixed in 4% paraformaldehyde for 48 h, embedded in paraffin, and then were cut into 5-μm-thick sections. Afterward, sections were deparaffinized by xylene, hydrated by gradient ethanol, and then stained with periodic acid-Schiff (PAS). The sections were observed under a light microscopy (BX53, Olympus, Tokyo, Japan).

### Transmission Electron Microscopy

Fresh kidney tissues in 1 mm^3^ were fixed in 2.5% glutaraldehyde for 24 h and 1% leleonic acid for 2 h. Next, the samples were dehydrated with a series of ethanol and acetone, immersed with acetone and propylene oxide. Each sample was embedded and then sliced with ultramicrotome. Subsequently, the sections were dyed with uranium acetate and lead citrate, and the ultrastructure of the podocytes and the number of mitochondrial autophagosomes in the CGN model rats were observed under a Hitachi transmission electron microscope (TEM) (HT770, Tokyo, Japan). The foot process width and glomerular basement membrane thickness were calculated by using the ImageJ 1.48 software.

### Real-Time PCR Analysis

The total RNA was isolated from renal cortex homogenate using RNAiso Plus (Takara, Beijing, China) under the instructions of the manufacturer, and then, cDNA was synthesized by Prime Script RT Reagent Kit with gDNA Eraser (Takara, Beijing, China). Quantitative analysis of mitochondrial catalase (CAT), superoxide dismutase 2 (SOD2), and peroxiredoxin 3 (PRDX3) mRNAs were measured using the CFX96 Bio-Rad real-time PCR instrument (Berkeley, CA, United States) with TB Green Premix Ex Taq Kit (Takara, Beijing, China). The mRNA expression levels were normalized to GAPDH through the 2^−ΔΔCt^ method. All the primers were designed and synthesized by Sangon Biotech (Shanghai) Co., Ltd. The primer sequences used were as follows: CAT, 5′-CTG​ACT​GAC​GCG​ATT​GCC​TA-3′ and 3′-GTG​GTC​AGG​ACA​TCG​GGT​TT-5′; SOD2, 5′-CAC​CGA​GGA​GAA​GTA​CCA​CG-3′ and 3′-TGG​GTT​CTC​CAC​CAC​CCT​TA-5′; PRDX3, 5′-AGT​GTG​GAA​GAA​CCA​CTC​CG-3′ and 5′-TGG​CTT​GAT​CGT​AGG​GGA​CT-3′; GAPDH, 5′-ACA​GCA​ACA​GGG​TGG​TGG​AC-3′ and 3′-TTT​GAG​GGT​GCA​GCG​AAC​TT-5′.

### Cell Culture and Treatment

Immortalized mouse podocytes were cultured for 7 days in RPMI 1640 medium containing 10% FBS, 100 U/ml penicillin, 100 mg/ml streptomycin, and 10 U/ml interferon-γ (IFN-γ, PeproTech, Rocky Hill, NJ, United States) at 33°C and 5% CO_2_. Proliferated podocytes were transferred to 37°C in IFN-γ-free medium for 14 days. The subsequent experiment was conducted with well-differentiated podocytes. ZWT-containing serum and normal serum from healthy male SD rats were prepared as in a previous study (Li et al., 2020b). The podocytes were treated with 40 ng/ml of tumor necrosis factor-α (TNF-α) for 24 h in the absence or presence of ZWT-containing serum as follows: (Global, 2020) Control group (10% normal serum), (Floege and Amann, 2016) TNF-α group (40 ng/ml TNF-α + 10% normal serum), (Chadban and Atkins, 2005) 2.5% ZWT group (40 ng/ml TNF-α + 2.5% ZWT-containing serum + 7.5% normal serum), (Voskamp et al., 2017) 5% ZWT group (40 ng/ml TNF-α + 5% ZWT-containing serum + 5% normal serum), (Jefferson, 2018) 10% ZWT group (40 ng/ml TNF-α + 10% ZWT-containing serum). For some experiments, 20 μm PI3K inhibitor LY294002 (ApexBio Technology, Boston, MA, United States) or 10 μm PI3K agonist insulin-like growth factor-1 (IGF-1, PeproTech, Rocky Hill, NJ, United States) was treated together with 10% ZWT-containing serum.

### Measurement of ATP Content

Intracellular ATP content was detected using an ATP assay kit (Beyotime, Biotechnology, Shanghai, China). First, podocytes treated above were lysed, and the supernatant was obtained after being centrifuged at 12,000 × *g*, 4°C for 5 min. The ATP standard substance was diluted into 0.01, 0.03, 0.1, 0.3, 1, 3, and 10 μM. Next, 100 μl of ATP working solution was added into a 96-well plate, and then 20 μl of podocyte lysis or standard substance was added, respectively. After incubation in the dark for 5 min, the luminescence intensity of each sample was measured by a Tecan M1000 Pro plate reader (Männedorf, Switerland). The ATP levels were calculated and expressed as μM in reference to the corresponding standard curves.

### Mitochondrial Membrane Potential

Mitochondrial membrane potential (MMP) was assessed using a JC-1 staining kit (Yeasen Biotechnology, Shanghai, China) in accordance with the instructions of the manufacturer. Briefly, podocytes were washed with phosphate buffer saline (PBS) three times and incubated with JC-1 dyeing solution for 20 min at 37°C in the dark. Subsequently, the cells were rinsed with PBS thrice and then observed by a confocal laser microscope (LSM 800, ZEISS, Germany) at ×400 magnification. The green fluorescence (Ex/Em = 514/529 nm) was used for monomer detection and the red fluorescence (Ex/Em = 585/590 nm) for aggregates. Five randomly chosen fields were photographed and calculated with the ratio of aggregates/monomers for fluorescence intensity analysis by using ImageJ 1.48 software.

### Mitochondrial ROS Detection

The mitoSOX red fluorophore dye (Invitrogen, Carlsbad, CA, United States) was used for the determination of mtROS accumulation. Upon treatment with TNF-α or ZWT-containing serum, podocytes were ringed with Hank’s balanced salt solution (HBSS) thrice and then incubated with 2.5 μM mitoSOX working solution at 37°C for 10 min with light protection. After being washed with HBSS three times, podocytes were observed by a confocal laser microscope (LSM 800, ZEISS, Germany) at ×400 magnification. Five randomly chosen fields were photographed, and the fluorescence intensity of mitoSOX was analyzed by using the ImageJ 1.48 software.

### Immunofluorescence Analysis

Renal frozen sections, 4-μm thick, and cultured podocytes were fixed with paraformaldehyde at 4°C for 10 min, and then washed with PBS three times. Then samples were permeabilized with 0.5% Triton X-100 for 10 min, and blocked with normal goat serum at 37°C for 30 min. Next, all samples were incubated with LC3B (Abcam, Cambridge, United Kingdom) together with COX IV (Abcam) at 4°C overnight. After being washed with PBS, the sections or cells were incubated with goat-anti rabbit AF555 and goat-anti mouse AF488 antibodies purchased from Cell Signaling Technology (Beverly, MA, United States) for 30 min at 37°C in the dark. Samples were added with DAPI (5 μg/ml) for 5 min at room temperature. The sections and cells were washed with PBS three times and then observed under a confocal laser microscope (LSM 800, ZEISS, Germany) at ×400 magnification. Five randomly chosen fields were photographed and calculated with the area ratio of colocalized LC3 to COX IV in glomerulus analyzed by using the ImageJ 1.48 software.

### Western Blot Analysis

The protein extracts of renal cortex and podocytes were prepared in RIPA lysis buffer containing phosphatase inhibitors and protease (CoWin Biosciences, Beijing, China). Equal amounts of 30 μg of proteins were separated on 10% or 15% sodium dodecyl sulfate-polyacrylamide (SDS-PAGE) and transferred to polyvinylidene difluoride membranes (Millipore, Bedford, MA, United States). After that, the membranes were blocked with 5% bovine serum albumin (BSA) for 2 h at room temperature. Subsequently, membranes were incubated with corresponding primary antibodies at 4°C overnight as follows: LC3B (ab192890), p62 (ab56416) obtained from Abcam (Cambridge, United Kingdom), HSP60 (#12165S), TOMM20 (#42406S), PI3K (#4257S), p-AKT (#4060S), AKT (#4691S), p-AMPKα (#2535S), AMPKα (#2532), p-mTOR (#2971S) and mTOR (#2983S) purchased from Cell Signaling Technology (Beverly, MA, United States), GAPDH (D2817) from Santa Cruz (CA, United States). After being washed thrice with Tris-buffered saline Tween-20 (TBST), the membranes were incubated with secondary antibody conjugated with horseradish peroxidase purchased from Jackson Immuno Research (West Grove, PA, United States) at room temperature for 1 h. Finally, these membranes were washed again with TBST and detected using an enhanced chemiluminescence kit (Thermo Fisher Scientific, Boston, MA, United States). Quantification of the protein bands were analyzed using Image J 1.48 software.

### Statistical Analysis

All data were analyzed using SPSS 20.0 (SPSS, Inc., Chicago, IL, United States) and expressed as mean ± standard deviation (SD). The differences between groups were analyzed by one-way ANOVA. When *p* < 0.05, the differences were defined as statistically significant.

## Results

### Determination of the Main Chemical Constituents in Zhen-wu-tang

**FIGURE 1:**
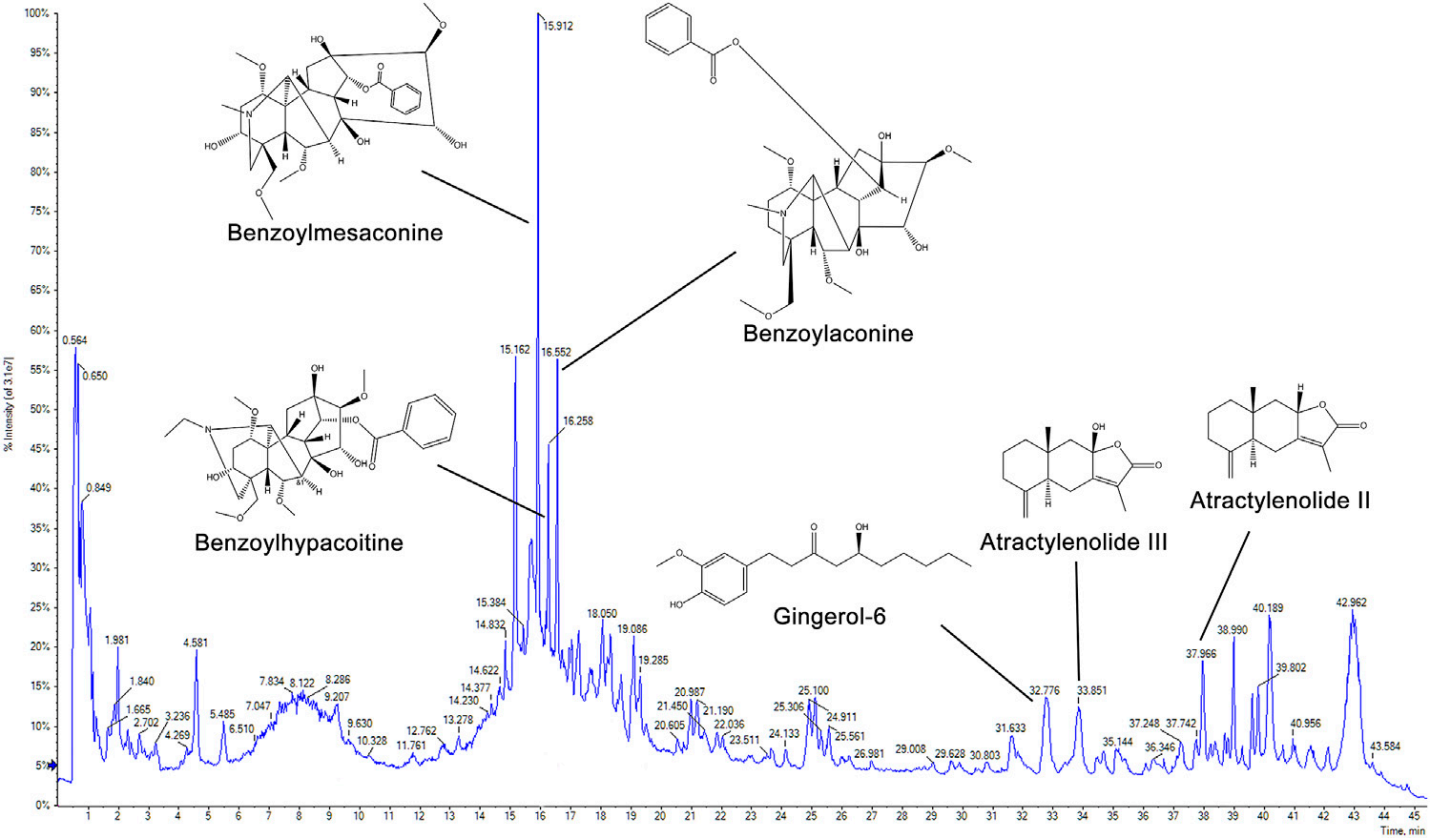
Chromatogram of Zhen-wu-tang (ZWT) by UPLC-Q-TOF-MS. The chemical structures of six marker ingredients are shown.

For quality assessment, the main chemical components of ZWT were identified with UPLC-Q-TOF-MS. As shown in Figure 1, six marker ingredients were separated within 45 min. The retention times of benzoylmesaconine, benzoylaconine, benzoylhypacoitine, gingerol-6, atractylenolide III, and atractylenolide II were detected at 15.912, 16.258, 16.552, 32.776, 33.851, and 37.966 min, respectively.

### Zhen-wu-tang Protected Kidney Injury in Chronic Glomerulonephritis Model Rats

**FIGURE 2:**
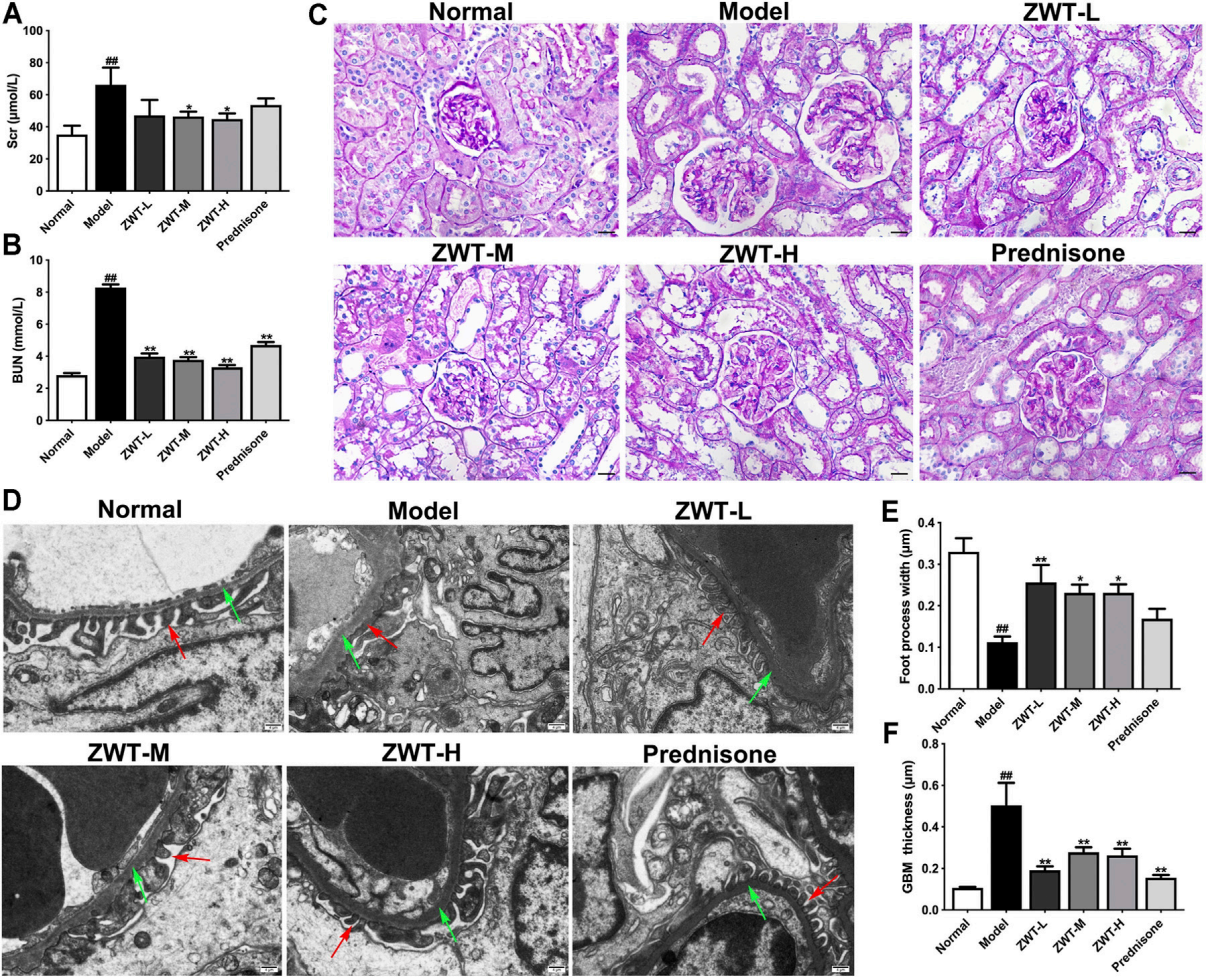
ZWT protected renal function and structural changes in chronic glomerulonephritis (CGN) model rats. **(A,B)** The levels of blood urea nitrogen (BUN) and serum creatinine (Scr). **(C)** Representative images of PAS-stained renal sections (×400 magnification; scale bar = 20 μm). **(D)** Representative images of renal tissue ultrastructure by transmission electron microscopy (×5,000 magnification; scale bar = 5 μm). Red arrows indicate foot process. Green arrows indicate glomerular basement membrane (GBM). **(E,F)** Quantitative analysis of foot process width and GBM thickness. Data are represented as mean ± SD (*n* = 8). ^##^ *p* < 0.01 versus normal group, *p < 0.05 and **p < 0.01 versus model group. ZWT ameliorated mitochondrial dysfunction in CGN model rats.

Serum creatinine (Scr) and blood urea nitrogen (BUN) are important indictors of renal function. As expected, in comparison with the model group, the levels of Scr and BUN were significantly decreased after ZWT treatment (Figures 2A,B). Next, the renal morphology was examined by PAS staining and Transmission electron microscopy (TEM). Our results showed that ZWT suppressed abnormal increase in mesangial matrix in glomeruli (Figure 2C), as well as ameliorated the foot process fusion and glomerular basement membrane (BGM) thickening (Figures 2D–F). Together, these results indicated that ZWT protected kidney injury in CGN model rats.

### Zhen-wu-tang Ameliorated Mitochondrial Dysfunction in Chronic Glomerulonephritis Model Rats

**FIGURE 3:**
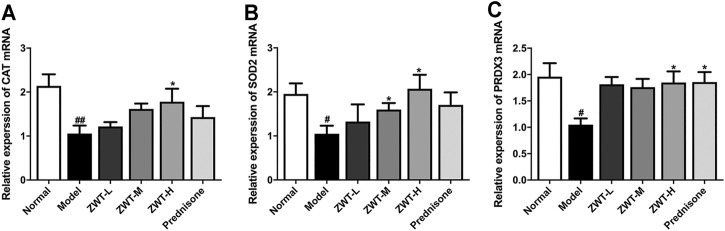
ZWT ameliorated mitochondrial dysfunction in CGN model rats **(A–C)**. The mRNA expressions of mitochondrial antioxidant enzymes mitochondrial catalase (CAT), superoxide dismutase 2 (SOD2), and peroxiredoxin 3 (PRDX3) in CGN model rats by RT-PCR. Data are represented as mean ± SD (*n* = 8). ^#^ *p* < 0.05, ^##^ *p* < 0.01 versus normal group, *p < 0.05 and **p < 0.01 versus model group. ZWT induced mitophagy in CGN model rats.

Mitochondrial dysfunction is involved in the pathogenesis of kidney diseases (Tang et al., 2021). As antioxidant defense is one of the main function of mitochondria, the mRNA levels of mitochondrial antioxidant enzymes, including catalase (CAT), superoxide dismutase 2 (SOD2) and peroxiredoxin 3 (PRDX3), were measured. Real-time PCR results showed that ZWT remarkably increased the mRNA expressions of antioxidant enzymes compared with the CGN model group, which manifested that ZWT ameliorated mitochondrial dysfunction in CGN rats (Figure 3).

### Zhen-wu-tang Induced Mitophagy in Chronic Glomerulonephritis Model Rats

**FIGURE 4:**
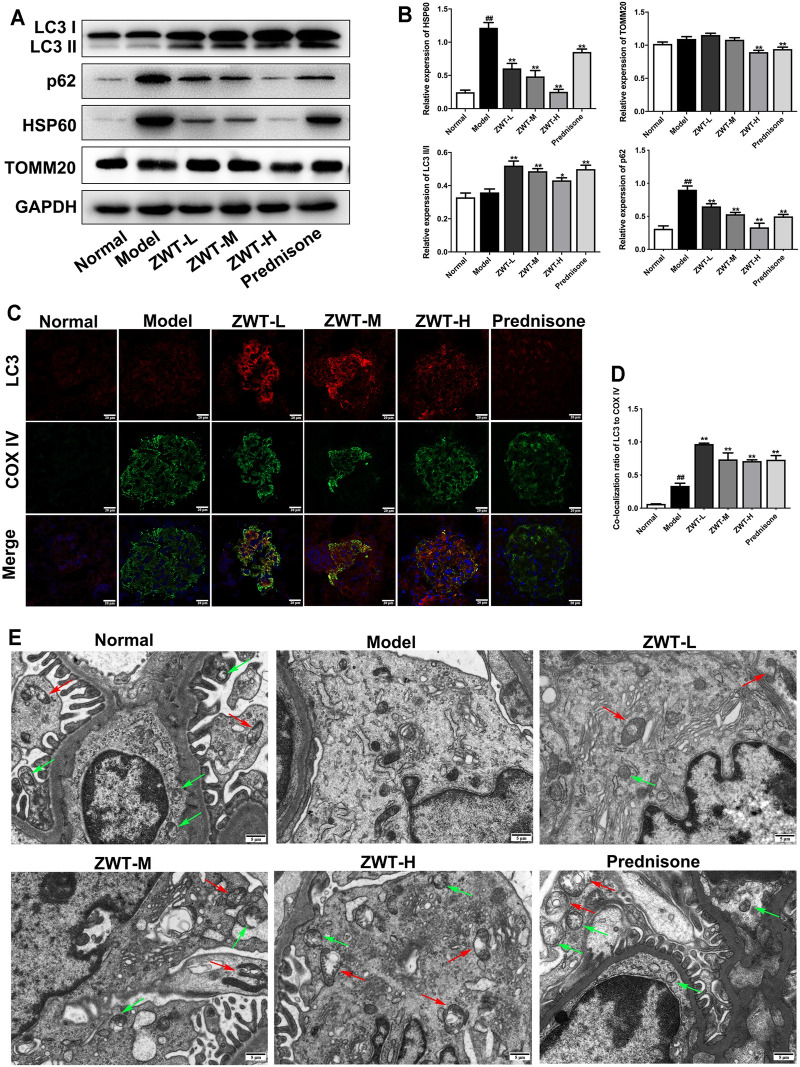
ZWT induced mitophagy in CGN model rats. **(A, B)** The protein blots and quantitative analysis of LC3 II/I, p62, heat shock protein 60 (HSP60), and translocase of outer mitochondrial membrane 20 (TOMM20). The obtained values of HSP60, TOMM20, and p62 were normalized to GAPDH; LC3 II values were normalized to LC3 I. **(C, D)** The representative images and quantitative analysis of LC3 and COX IV colocalization (×400 magnification; scale bar = 20 μm). **(E)** Representative images of mitochondrial autophagosomes by Transmission electron microscopy (×5,000 magnification; scale bar = 5 μm). Red arrows indicated mitochondrial autophagosome. Green arrows indicate autophagosome. Data are represented as mean ± SD (*n* = 3). ^##^ *p* < 0.01 versus normal group, *p < 0.05 and **p < 0.01 versus model group. ZWT alleviated mitochondrial dysfunction in podocytes.

Mitophagy is an important quality control mechanism for cell homeostasis by eliminating damaged mitochondria. To evaluate the regulatory effects of ZWT on mitophagy in CGN model rats, the expressions of autophagy-related proteins LC3 and p62, and mitochondrial proteins TOMM20 and HSP60, were detected. As shown in Figures 4A,B, the expression levels of LC3 II/I were increased, as p62, HSP60, and TOMM20 were decreased under ZWT administration. In addition, ZWT significantly induced the colocalization of LC3 and mitochondrial marker COX IV in the glomeruli (Figures 4C,D). Moreover, mitochondrial autophagosomes captured by TEM indicated that ZWT increased the number of autophagosomes and mitochondrial autophagosomes in kidney tissue (Figure 4E). Taken together, ZWT induced mitophagy in the CGN model rat kidneys.

### Zhen-wu-tang Alleviated Mitochondrial Dysfunction in Podocytes

**FIGURE 5:**
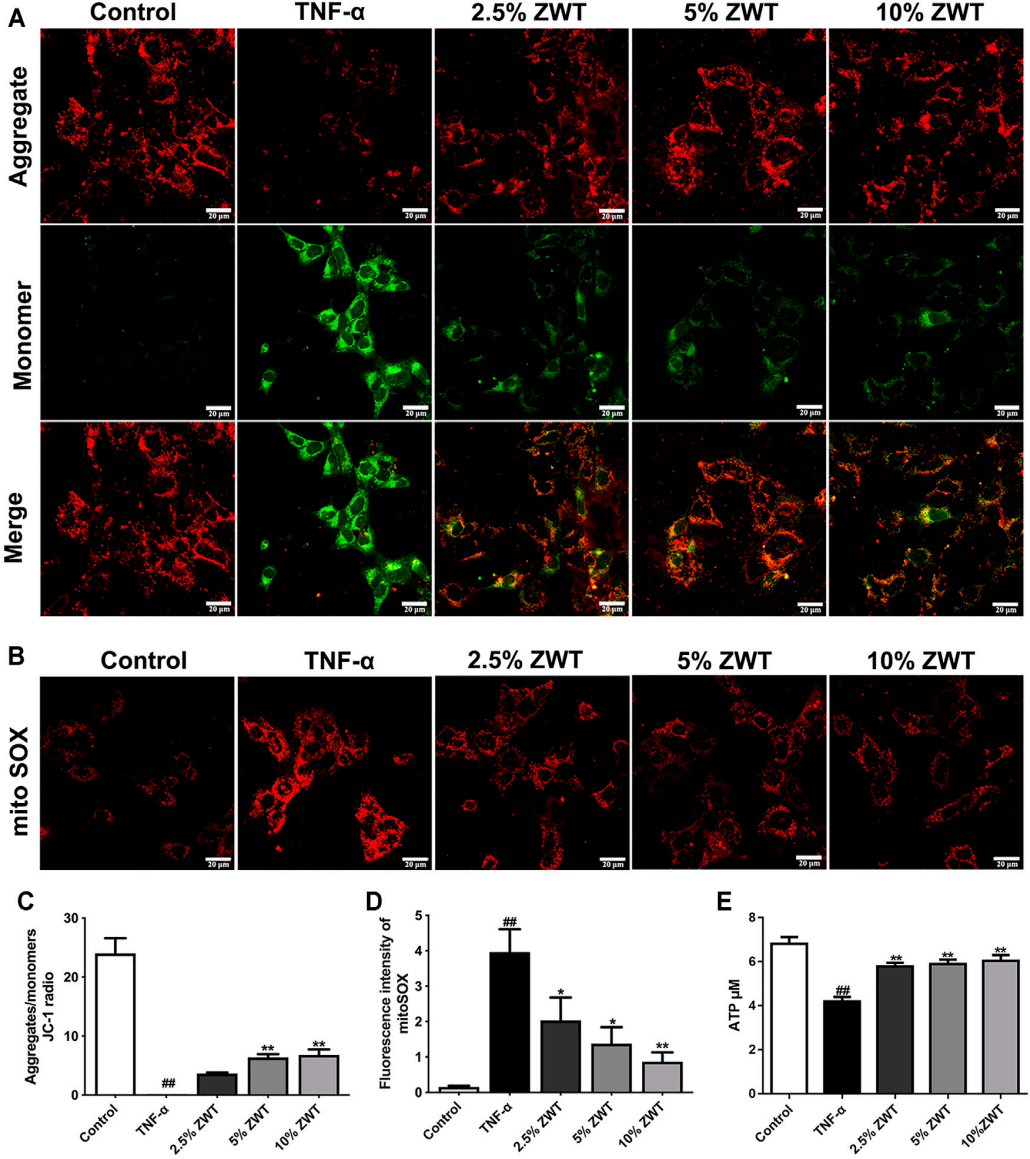
ZWT alleviated mitochondrial dysfunction in podocyte. **(A)** Representative images of mitochondrial membrane potential in podocytes (×400, scale bar = 20 μm). **(B)** Representative images of mitoSOX in podocytes (×400, scale bar = 20 μm). **(C)** The ratio of aggregates/monomers of JC-1 probe. **(D, E)** The fluorescence intensity of mitoSOX, ATP levels in podocytes. Data are expressed as mean ± SD, *n* = 3. ^#^ *p* < 0.05, ^##^ *p* < 0.01 versus control group, *p < 0.05 and **p < 0.01 versus TNF-α group. ZWT promoted mitophagy in podocytes.

To assess the effect of ZWT on mitochondrial function *in vitro*, mouse podocytes were simulated with TNF-α and treated with different proportions of ZWT-containing serum. Mitochondrial membrane potential (MMP) was detected by JC-1 probe. The findings showed that the ratio of aggregates/monomers increased under ZWT treatment, suggesting that the MMP was returned to normal (Figures 5A, C). MitoSOX is a specific fluorescent probe for mitochondrial ROS (mtROS) assessment. ZWT treatment dramatically suppressed mtROS accumulation in podocytes (Figures 5B, D). Besides, as shown in Figure 5E, intracellular ATP concentration was decreased in response to TNF-α, while it was reversed by treating with ZWT. Overall, ZWT effectively alleviated mitochondrial dysfunction in damaged podocytes induced by TNF-α.

### Zhen-wu-tang Promoted Mitophagy in Podocytes

**FIGURE 6:**
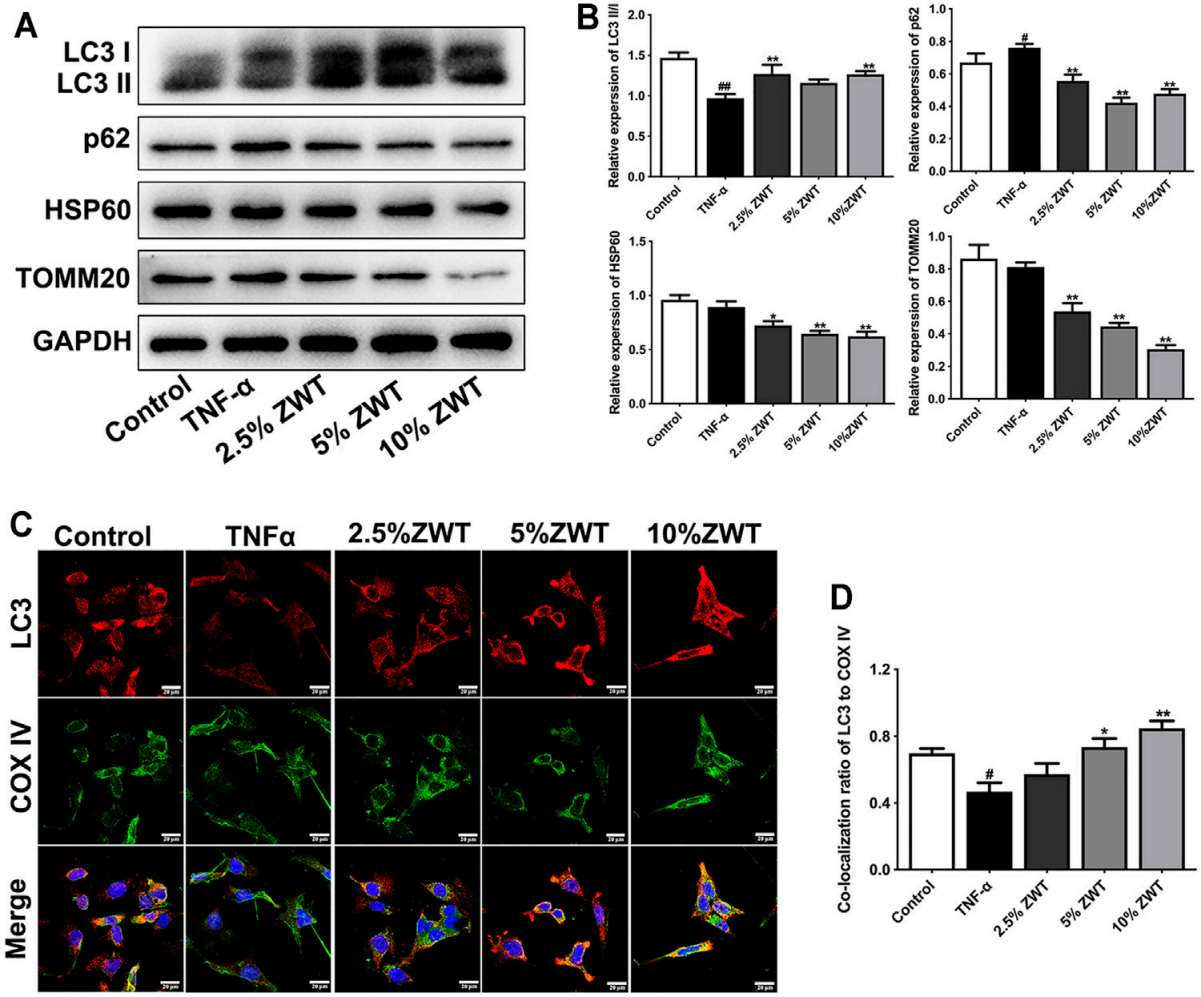
ZWT promoted mitophagy in podocytes. **(A, B)** The protein blots and quantitative analysis of LC3 II/I, p62, HSP60, and TOMM20. The obtained values of HSP60, TOMM20, and p62 were normalized to GAPDH; LC3 II values were normalized to LC3 I. **(C, D)** The representative images and quantitative analysis of LC3 and COX IV colocalization (×400, scale bar = 20 μm). Data are represented as mean ± SD (*n* = 3). ^#^ *p* < 0.05, ^##^ *p* < 0.01 versus control group, *p < 0.05 and **p < 0.01 versus TNF-α group.

Inconsistent with the study *in vivo*, the expressions and colocation of mitophagy-related proteins were determined in damaged podocytes as well. As shown in Figures 6A,B, the decreased expressions of LC3 II/I and increased levels of p62 in TNF-α-induced podocytes were reversed by ZWT. Besides, the expressions of HSP60 and TOMM20 were decreased under the treatment of ZWT-containing serum. In addition, ZWT markedly increase the colocalization of LC3 and COX IV in podocytes (Figures 6C,D). These results suggested that ZWT promoted mitophagy in damaged podocytes.

### Zhen-wu-tang Regulated PI3K/AKT/mTOR and AMPK Pathways in Chronic Glomerulonephritis

**FIGURE 7:**
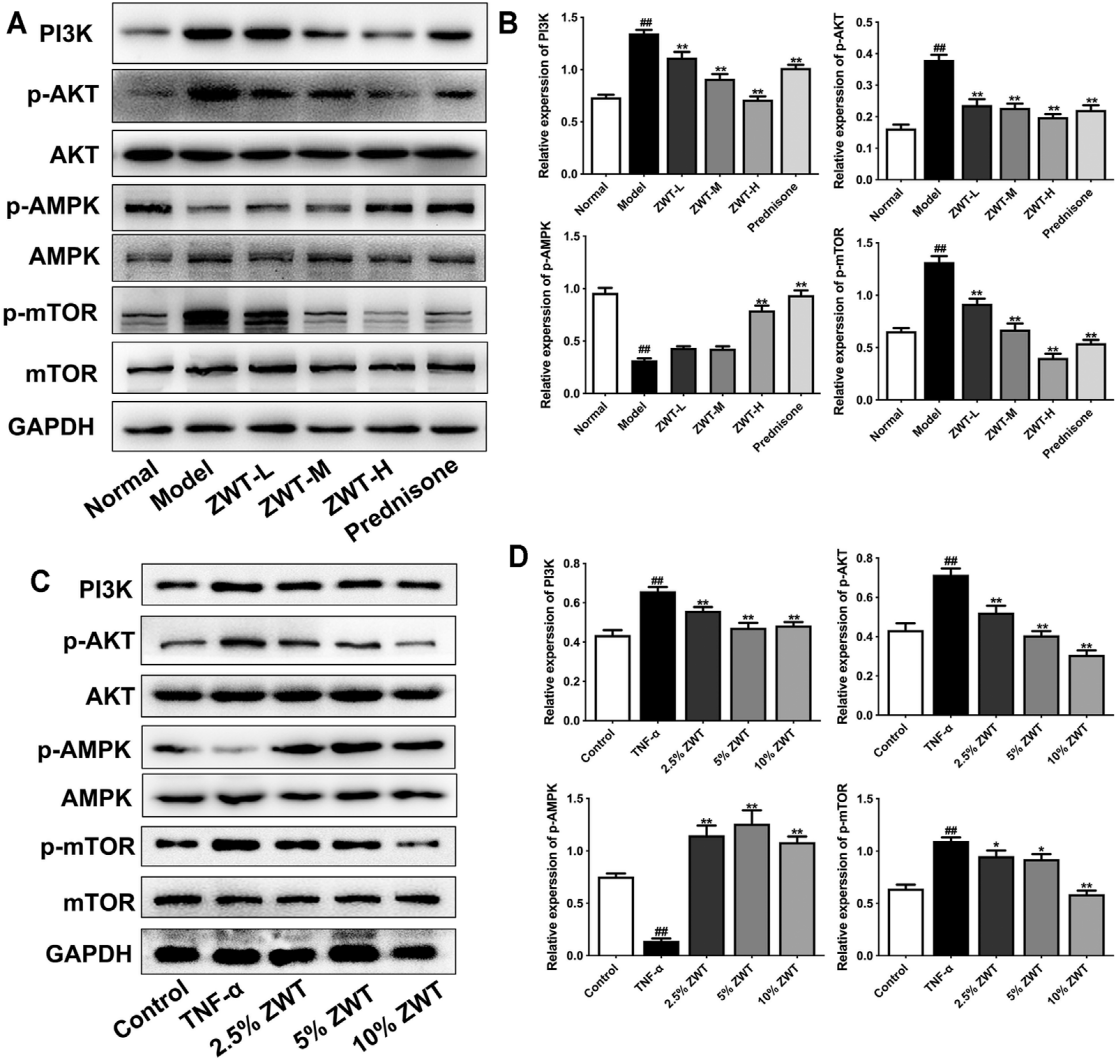
ZWT regulated the phosphatidylinositol 3-kinase (PI3K)/AKT/mTOR and AMPK pathways in CGN. **(A,B)** The protein blots and quantitative analysis of PI3K, p-AKT, p-AMPK, and p-mTOR in renal tissue. **(C,D)** The protein blots and quantitative analysis of PI3K, p-AKT, p-AMPK, and p-mTOR in podocytes. The obtained values of PI3K were normalized to GAPDH, p-AKT values were normalized to AKT, p-AMPK values were normalized to AMPK, and p-mTOR values were normalized to mTOR. Data are represented as mean ± SD (*n* = 3). ^#^ *p* < 0.05, ^##^ *p* < 0.01 versus normal or control group, *p < 0.05 and **p < 0.01 versus the model group or TNF-α group. The intervention of PI3K inhibitor and agonist against ZWT.

It is well known that the phosphatidylinositol 3-kinase (PI3K)/AKT/mTOR pathway has inhibitory roles on the autophagic pathway. Recently, some studies suggested that the PI3K/AKT/mTOR pathway is also involved in mitophagy (Maiti et al., 2019). Furthermore, the activation of AMPK pathway promoted mitophagy by enhancing mitochondrial fission and autophagosomal engulfment (Seabright et al., 2020). Therefore, in this study, we explored whether ZWT could regulate the PI3K/AKT/mTOR and AMPK pathways in CGN. As shown in Figures 7A,B, the expressions of PI3K, p-AKT, and p-mTOR were increased in CGN model rats compared with the control group. ZWT and prednisone significantly inhibited the activation of the PI3K/AKT/mTOR pathway. On the other hand, ZWT-H dose and prednisone reversed the decreased expression of p-AMPK in CGN rats. In podocytes, Western blot results showed that TNF-α upregulated the proteins in the PI3K/AKT/mTOR pathway and inhibited the AMPK pathway. Luckily, the ZWT-containing serum inhibited the expressions of PI3K, p-AKT, and p-mTOR, but upregulated the expression of p-AMPK (Figures 7C,D). These results demonstrated that ZWT induced mitophagy in CGN probably by inhibiting PI3K/AKT/mTOR and upregulating the AMPK pathway in some level.

### The Intervention of PI3K Inhibitor and Agonist Against Zhen-wu-tang

**FIGURE 8:**
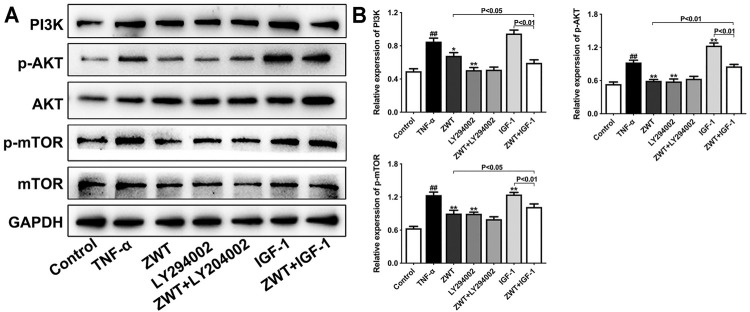
The intervention of PI3K inhibitor and agonist against ZWT. **(A,B)** The protein blots and quantitative analysis of PI3K, p-AKT, and p-mTOR. The obtained values of PI3K were normalized to GAPDH; p-AKT values were normalized to AKT. p-mTOR values were normalized to mTOR. Data are represented as mean ± SD (*n* = 3). ^#^ *p* < 0.05, ^##^ *p* < 0.01 versus control group, *p < 0.05 and **p < 0.01 versus the TNF-α group.

PI3K inhibitor LY294002 and agonist insulin-like growth factor-1 (IGF-1) were used alone or with ZWT to intervene in podocytes in this study. As shown in Figure 8, LY294002 mostly reduced the protein expressions of PI3K, p-AKT, and p-mTOR compared with the TNF-α group, and ZWT combined with LY294002 significantly enhanced the inhibitory effect of ZWT on PI3K expression. Conversely, IGF-1 upregulated the PI3K/AKT/mTOR pathway in podocytes. More importantly, ZWT combined with IGF-1 could obviously downregulate the activation of the PI3K/AKT/mTOR pathway compared with ZWT. These results further confirmed the inhibitory effect of ZWT on the PI3K/AKT/mTOR pathway, which maybe an essential regulatory mechanism of ZWT on CGN therapy.

## Discussion

In the current study, we investigated the protective effects and regulatory mechanisms of ZWT in CGN. We found that ZWT protected kidney injury in CGN model rats. Furthermore, ZWT ameliorated mitochondrial dysfunction in renal tissue of CGN model rats and damaged podocytes induced by TNF-α. The mechanisms of ZWT above is related to inducing mitophagy through the PI3K/AKT/mTOR and AMPK pathways.

Mitochondria are classically described as energy-producing organelles through generation of adenosine triphosphate (ATP). Mitochondria also plays a critical role in oxidative phosphorylation, fatty acid oxidation, and amino acid catabolism (Chan, 2006). Mitochondrial disorder results from a mutant in the mitochondrial DNA or nuclear genes that impede mitochondrial function. Upon stress, impaired mitochondrial dynamics and incomplete mitochondrial membrane lead to the loss of membrane potential, mitochondrial permeability transition, reactive oxygen species (ROS) production, release of apoptogenic factors, and energetic failure to induce cell injury and death (Nunnari and Suomalainen, 2012). Human kidneys demand lots of energy for filtration of blood, regulation of blood pressure, reabsorption of nutrients, maintenance of fluid homeostasis and electrolytes. Not only that: renal function depends on interplay between multiple cell types including endothelial cells, podocytes, mesangial cells, and tubulointerstitial cells, which relies on regular mitochondrial function (Maezawa et al., 2015). Therefore, maintaining normal status of the mitochondria is central for kidney function. Imbalance in mitochondrial homeostasis has been implicated in the development and progression of CGN. Mitochondrial protection is an effective therapeutic strategy for various kinds of kidney diseases (Tang and Dong, 2016). Podocytes are terminally differentiated epithelial cells, which require a high energy demand to remodel foot processes in the glomerulus by maintaining cytoskeletal and extracellular matrix proteins. Podocyte injury or loss is the main reason of albuminuria and leads to progression of glomerular diseases (Gujarati et al., 2020). In the present study, ZWT protected against podocyte function by inhibiting the fusions of foot processes and incrassation of the basement membrane. Moreover, TNF-α damaged cultured mouse podocytes with decreased levels of ATP content and MMP, which were ameliorated by ZWT. These results suggested that ZWT suppressed mitochondrial dysfunction to protect podocyte injury in CGN.

Even though clinic application and our previous studies have proved the pharmacodynamics of ZWT on kidney diseases, there is a lack of information about the pharmacokinetics of ZWT. Some research demonstrated that the exact or the active components of ZWT improved mitochondrial function. Compounds mesaconitine, benzoylaconitine, and benzoylhypacoitine might be the principal active components of Fuzi for the main activities of energy metabolism of the mitochondria (Zheng et al., 2014). The major component of *Atractylodes macrocephala* Koidz, atractylenolide III ameliorated cerebral ischemic injury by inhibiting mitochondrial fission dependent on the JAK2/STAT3/Drp1 pathway (Zhou et al., 2019). Paeoniflorin, from *Paeonia lactiflora* Pall, protected SH-SY5Y cell injury by preventing mitochondrial dysfunction (Wang et al., 2014). The extract of *Zingiber officinale* Roscoe and its major active component 6-gingerol promoted mitochondrial biogenesis via the AMPK/PGC1-α signaling pathway (Deng et al., 2019). However, it is unclear what the active components of ZWT are for mitochondrial function regulation in CGN.

Oxidative stress is known as the main pathological cause of glomerulonephritis. The intracellular ROS mainly generated, while mitochondria are producing ATP via oxidative phosphorylation. Excess ROS triggers changes in mitochondrial structure including the opening of mitochondrial permeability transition pore, which result in mitochondrial dysfunction, organelle swelling, and eventual cell death (Zorov et al., 2014). As a result, damaged mitochondria, as the main source of ROS, produces more ROS, which causes a vicious cycle to aggravate kidney damage. Some researches proved that some mitochondria-targeted antioxidants, such as MitoQ, CoQ10, Mito-CP, and SkQ1, were beneficial in acute kidney diseases (Kezic et al., 2016). The concentrations of hydrogen peroxide and superoxide in the mitochondrial matrix are assessed mainly by rates of production, the activities of mitochondrial antioxidant enzymes, superoxide dismutase-2 (SOD2) and peroxiredoxin-3 (PRDX3), and catalase (CAT) (Brand, 2020). Therefore, the mRNA levels of SOD2, CAT, and PRDX3 in renal tissue of CGN rats were measured in this study. Results showed that the decrease in CAT, SOD2, and PRDX3 were significantly improved under ZWT administration. Moreover, ZWT-containing serum inhibited the accumulation of mtROS in damaged podocytes.

Mitophagy is a selective form of autophagy that eliminates redundant or damaged mitochondria (Wang and Klionsky, 2011). During mitophagy, mitophagy receptors bind certain ubiquitinated mitochondrial outer membrane proteins, such as translocase of the outer mitochondrial membrane 20 (TOMM20), leading to the proteasomal degradation of these mitochondrial proteins independent of microtubule-associated protein 1 light chain 3 beta (MAP1LC3B/LC3B) (Stolz et al., 2014). SQSTM1/p62 (p62), as mitochondrial outer membrane receptors, binds to LC3 to mediate mitophagy (Lazarou et al., 2015). It is reported that aberrant p62 affects the balance of mitophagy and further disturbs mitochondrial quality control (Liu et al., 2017). Under stressful conditions, mitophagy is induced as an adaptive or defense mechanism for maintaining mitochondrial function and thereby cell survival. So far, defective mitophagy has been implicated in the pathogenesis of a variety of human illnesses including neurodegenerative diseases, metabolic diseases, and cardiovascular diseases (Killackey et al., 2020). As mentioned above, mitochondrial injury contributes critically to abnormal kidney repair. Thus, timely removal of injured mitochondria may facilitate kidney damage. Our present study showed that ZWT induced mitophagy and macro-autophagy in CGN model rats by increasing the expression of LC3 together with mitochondrial complex IV protein (COX IV) in the glomerulus, as well as decreasing the expressions of p62, TOMM20, and heat shock protein 60 (HSP60). Meanwhile, ZWT increased the number of autophagosome and mitochondrial autophagosome in the renal cortex. Similarly, ZWT-containing serum improved mitophagy in podocytes simulated with TNF-α as well. These results suggested that ZWT ameliorated mitochondrial function in the kidneys possibly through inducing mitophagy.

The phosphatidylinositol 3-kinase (PI3K)/AKT/mammalian target of rapamycin (mTOR) signaling pathway plays an important role in the regulation of cell survival, growth, and proliferation. mTORC1 is considered a negative regulator of autophagy because suppression of mTORC1 activates autophagy (Kaushal et al., 2019). Downregulation of the PI3K/AKT/mTOR pathway protects against lipopolysaccharide-induced acute kidney injury by enhancing autophagy (Zhao et al., 2020). Recent studies manifested that mTOR inhibitor rapamycin improved mitochondrial dysfunction via lupus-prone mice (Oaks et al., 2016) and inhibits apoptosis by activating mitophagy in spinal ischemia–reperfusion injury (Li et al., 2018). Thioredoxin-interacting protein (TXNIP)-dependent activation of mTOR signaling pathway contributes to dysfunctional mitophagy in the diabetic kidneys (Huang et al., 2016). Consequently, the PI3K/AKT/mTOR signaling pathway may play a central role in regulating mitophagy in CGN. In the present study, we investigated the regulating effects of ZWT in the PI3K/AKT/mTOR pathway. Luckily, ZWT suppressed the increased protein expressions of PI3K, p-AKT, and p-mTOR in the CGN model rats and damaged podocytes. In addition, the regulatory effects of ZWT on the PI3K/AKT/mTOR pathway were disturbed by PI3K inhibitor LY294002 and agonist insulin-like growth factor 1 (IGF-1).

The AMP-activated protein kinase (AMPK) is a master regulator of metabolism, which is regulated by a wide array of metabolic stresses. Recent studies revealed that AMPK are involved in various aspects of mitochondrial homeostasis, such as mitophagy (Herzig and Shaw, 2018). In the present study, we also found that ZWT activated the AMPK pathway in CGN model rats and damaged podocytes in some degree, which indicated that ZWT not only regulated the PI3K/AKT/mTOR pathway but also adjusted the AMPK pathway in CGN.

In conclusion, our present study provided evidence that ZWT protects against kidney injury and podocyte injury in CGN model rats by ameliorating mitochondrial function. Furthermore, the above protective mechanisms of ZWT are irrelevant to the induction mitophagy via the PI3K/AKT/mTOR and AMPK pathways.

## Data Availability

The raw data supporting the conclusions of this article will be made available by the authors, without undue reservation.
